# Supplementary figures and images for: Identification of TAT as a Biomarker Involved in Cell Cycle and DNA Repair in Breast Cancer
Source: Biomolecules. 2024 Aug 30;14(9):1088. doi: 10.3390/biom14091088 (PMC11430390; doi:10.3390/biom14091088)

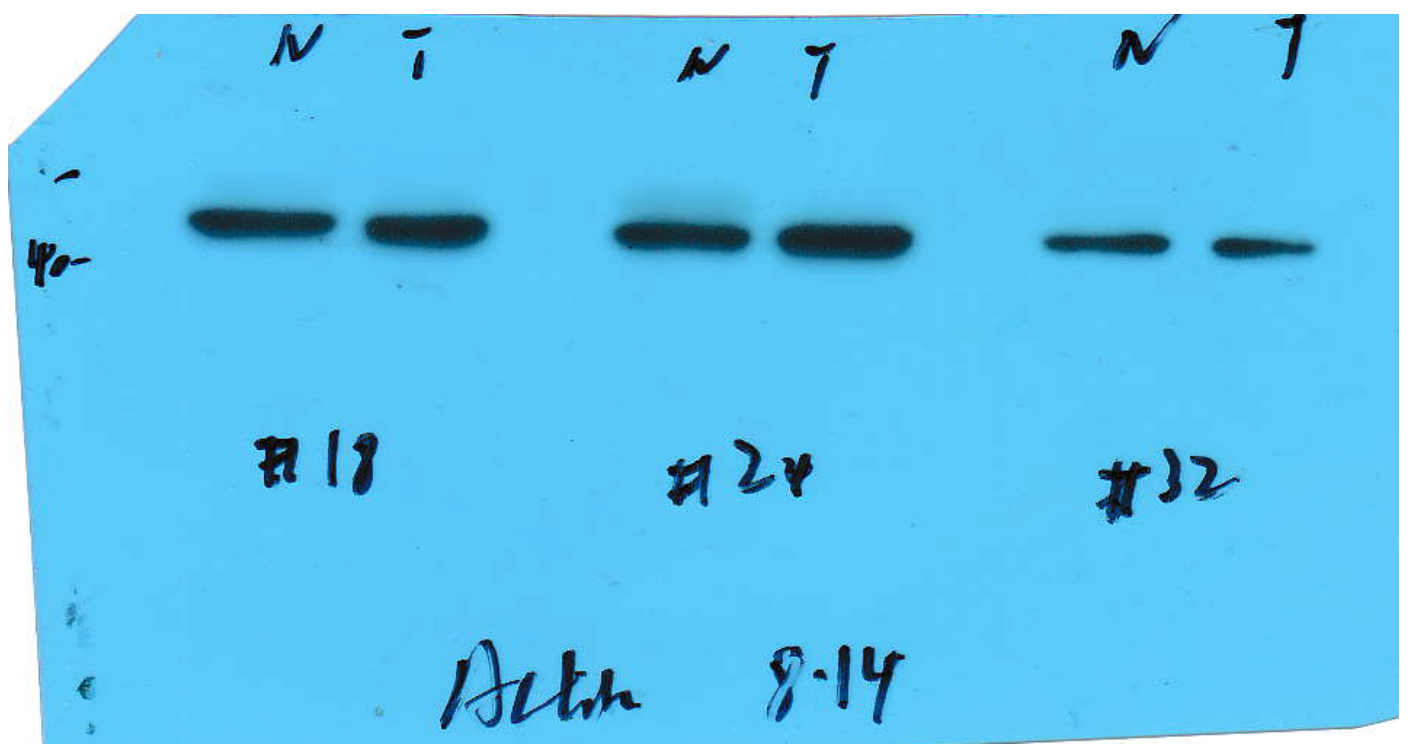

Supplement: Supplementary file 1 [file biomolecules-14-01088-s001.zip › Actin of 1-2.tif]

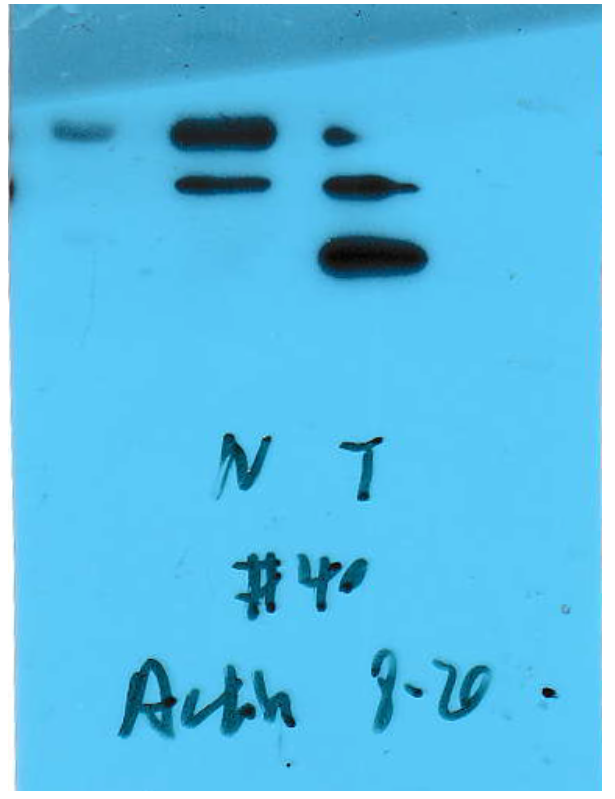

Supplement: Supplementary file 1 [file biomolecules-14-01088-s001.zip › Actin of 10.tif]

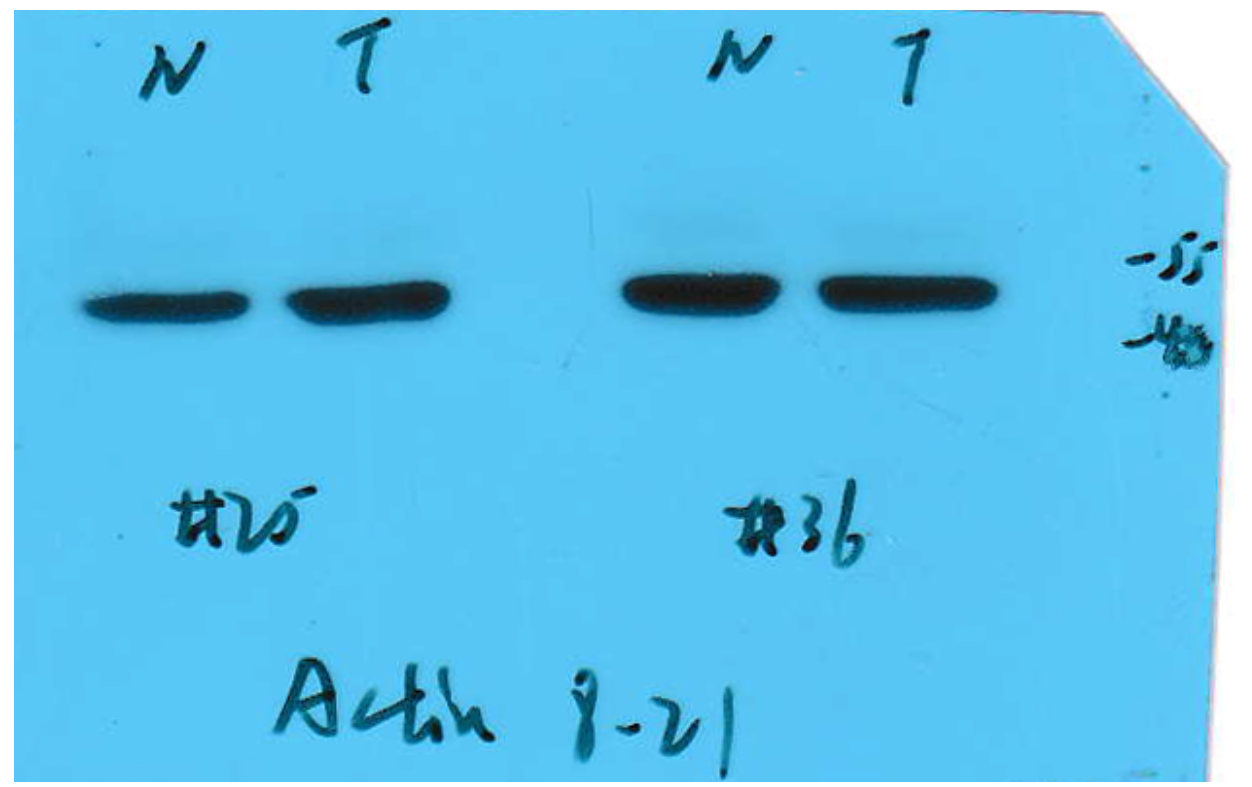

Supplement: Supplementary file 1 [file biomolecules-14-01088-s001.zip › Actin of 11-12.tif]

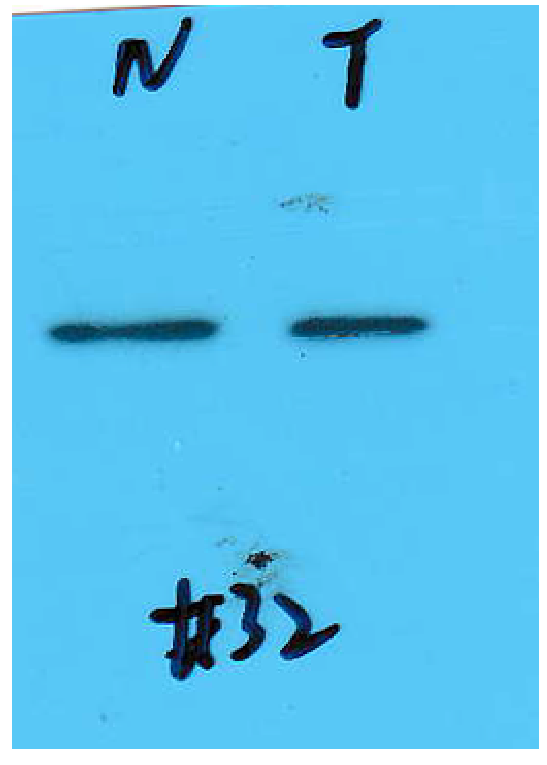

Supplement: Supplementary file 1 [file biomolecules-14-01088-s001.zip › Actin of 3.tif]

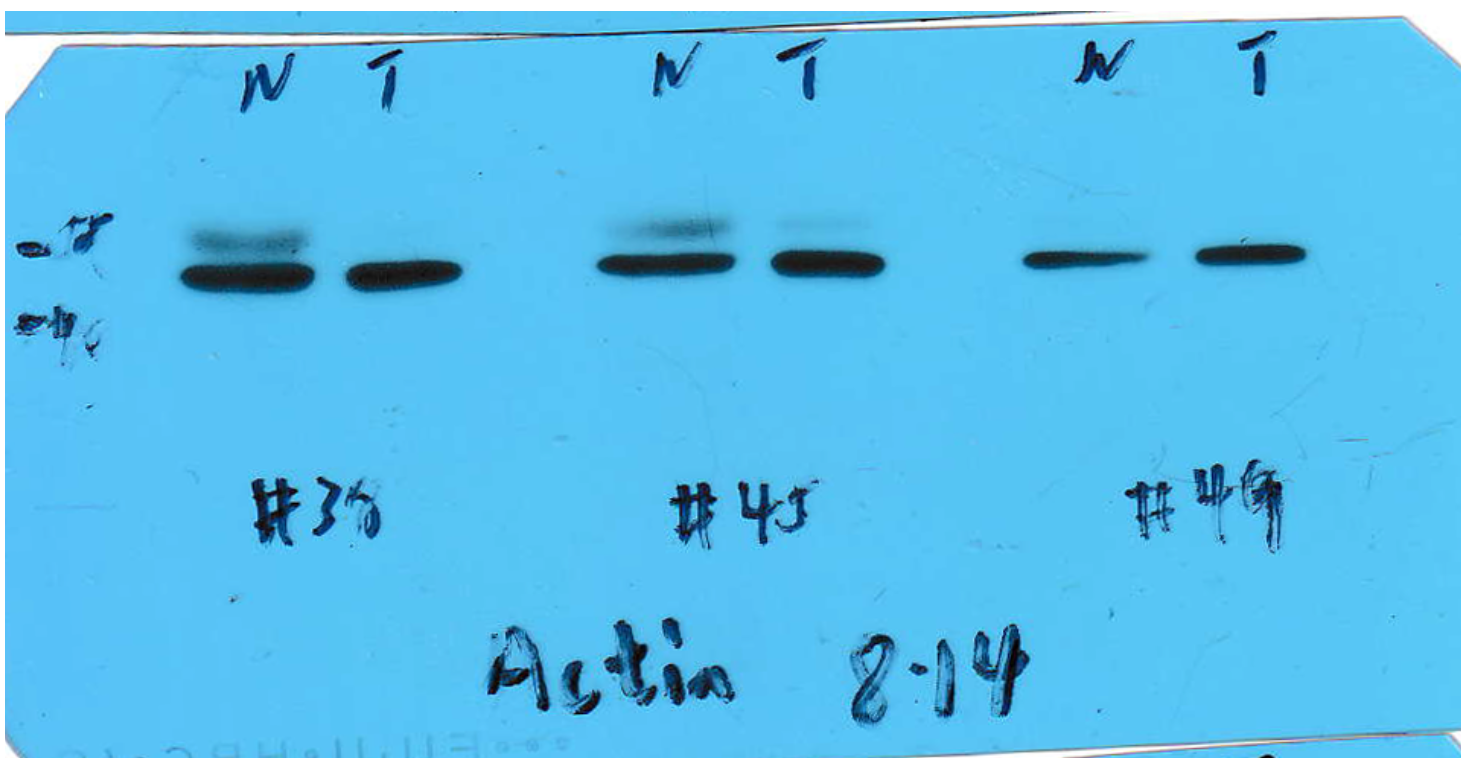

Supplement: Supplementary file 1 [file biomolecules-14-01088-s001.zip › Actin of 4-6.tif]

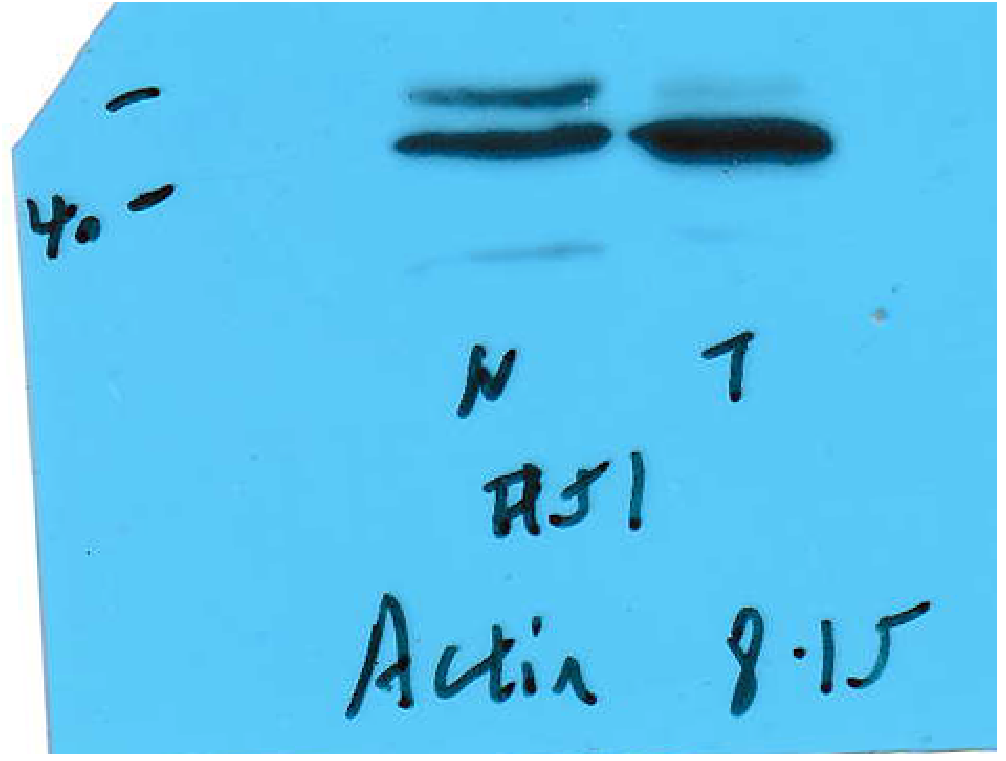

Supplement: Supplementary file 1 [file biomolecules-14-01088-s001.zip › Actin of 7.tif]

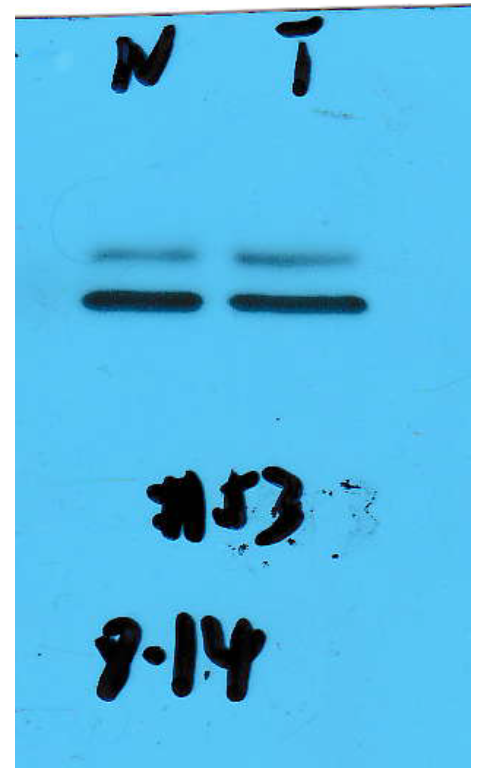

Supplement: Supplementary file 1 [file biomolecules-14-01088-s001.zip › Actin of 8.tif]

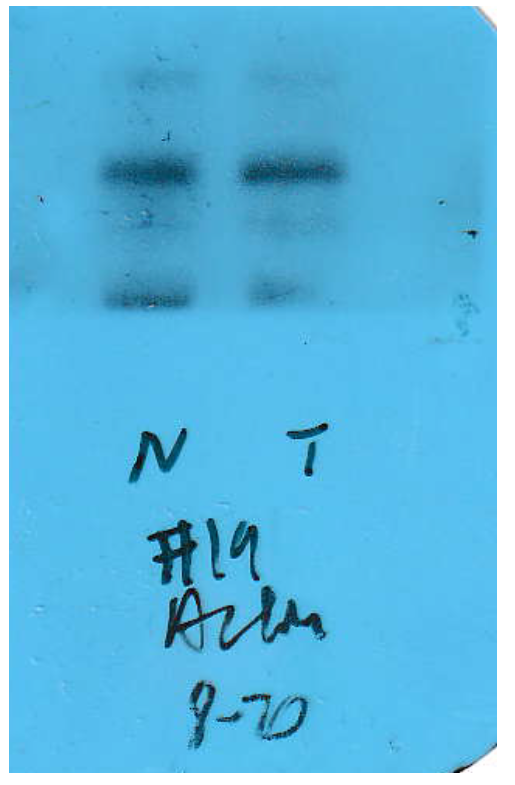

Supplement: Supplementary file 1 [file biomolecules-14-01088-s001.zip › Actin of 9.tif]

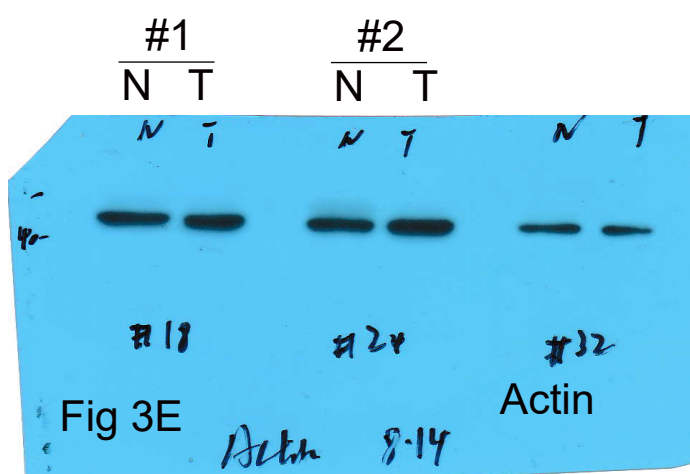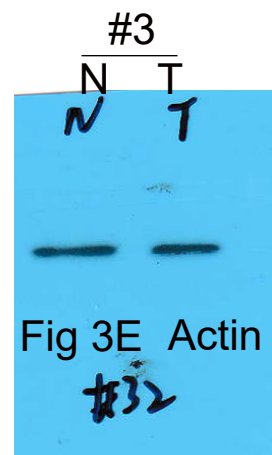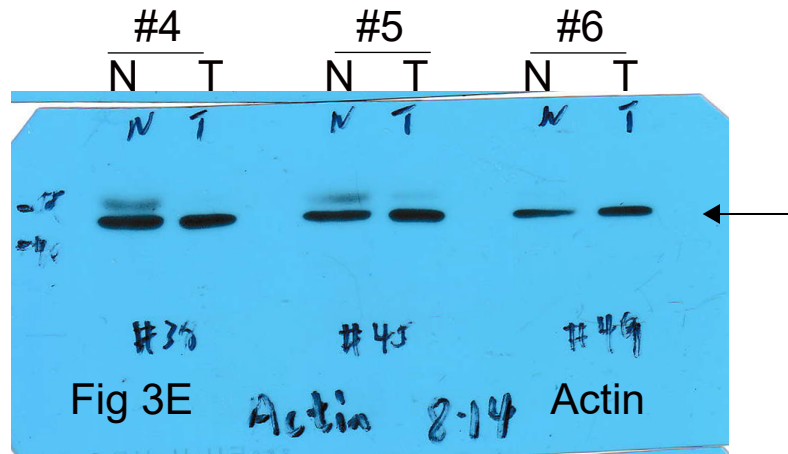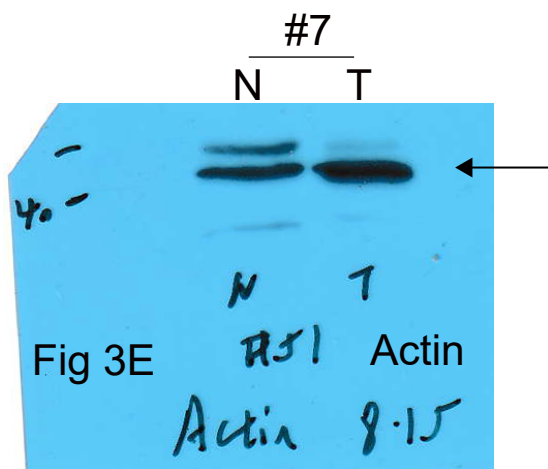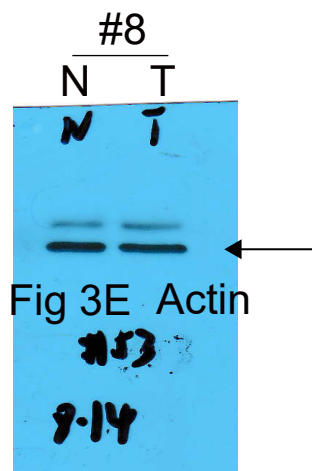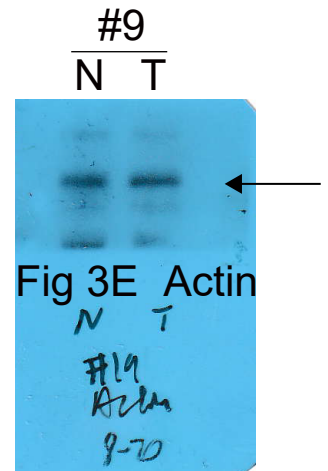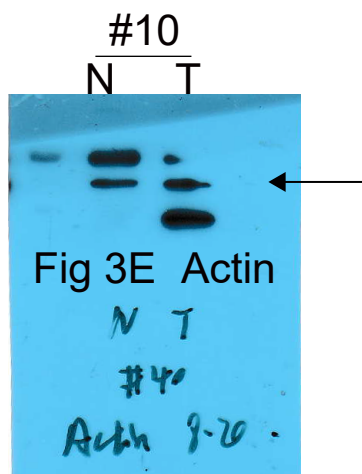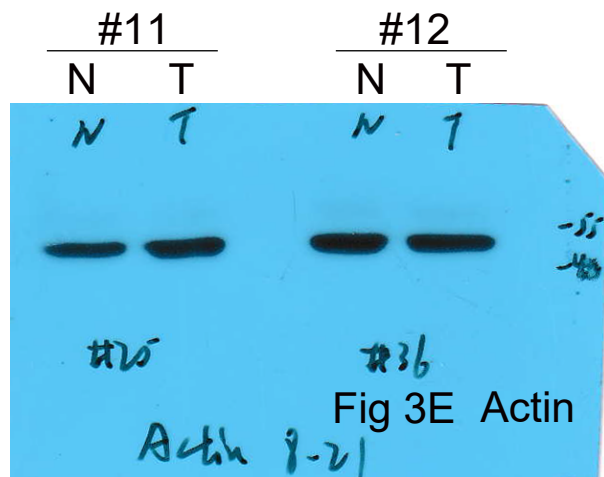

Supplement: Supplementary file 1 [file biomolecules-14-01088-s001.zip › Actin of Fig 3E.pdf]

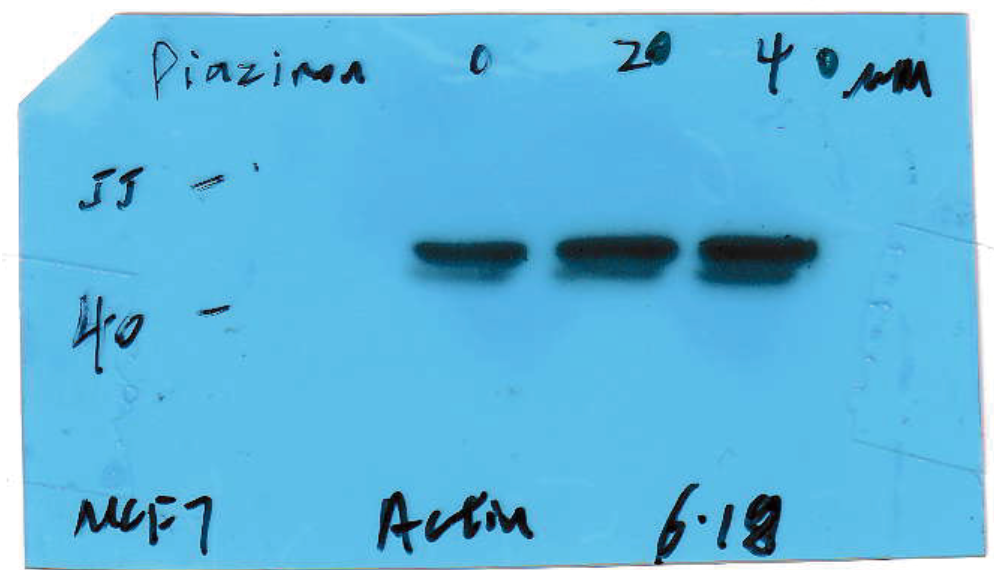

Supplement: Supplementary file 1 [file biomolecules-14-01088-s001.zip › Actin-MCF7.tif]

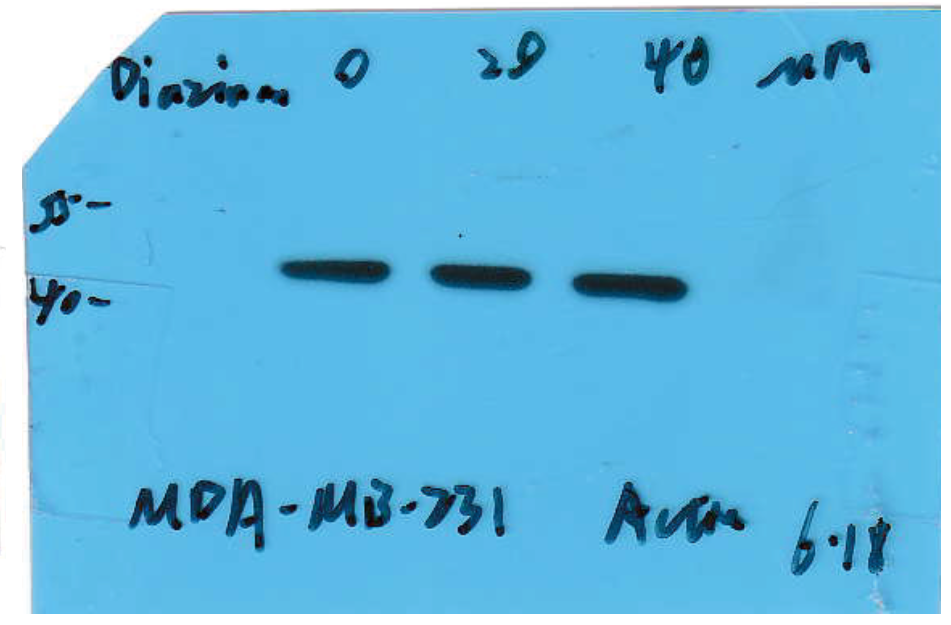

Supplement: Supplementary file 1 [file biomolecules-14-01088-s001.zip › Actin-MDA-MB-231.tif]

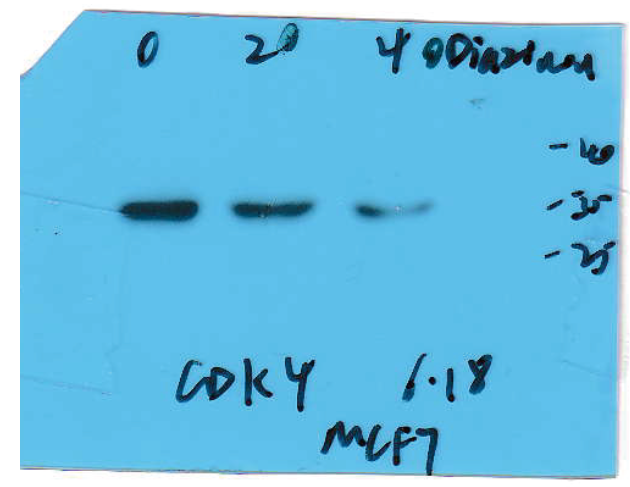

Supplement: Supplementary file 1 [file biomolecules-14-01088-s001.zip › CDK4-MCF7.tif]

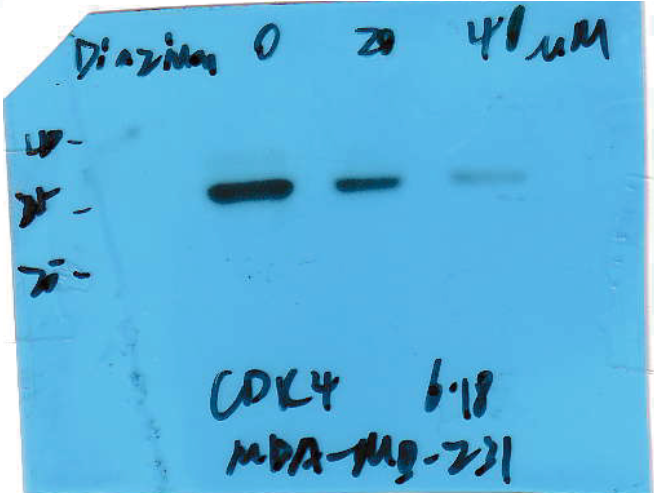

Supplement: Supplementary file 1 [file biomolecules-14-01088-s001.zip › CDK4-MDA-MB-231.tif]

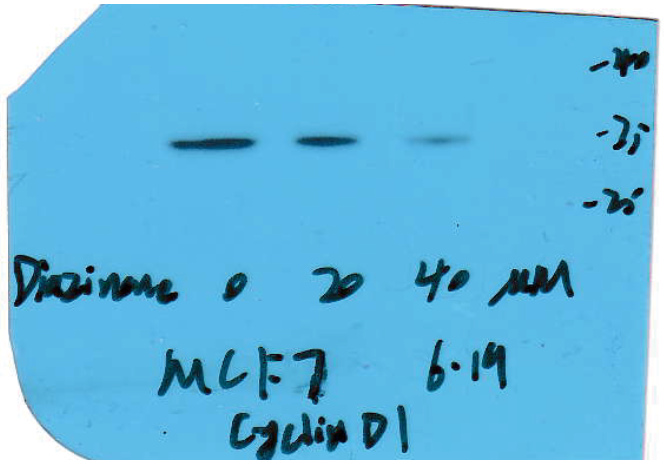

Supplement: Supplementary file 1 [file biomolecules-14-01088-s001.zip › cyclinD1-MCF7.tif]

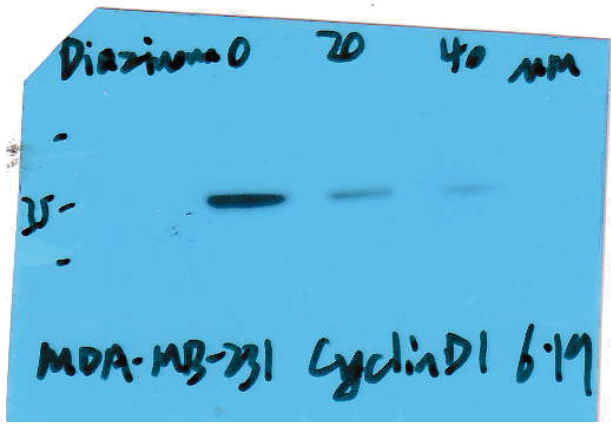

Supplement: Supplementary file 1 [file biomolecules-14-01088-s001.zip › cyclinD1-MDA-MB-231.tif]

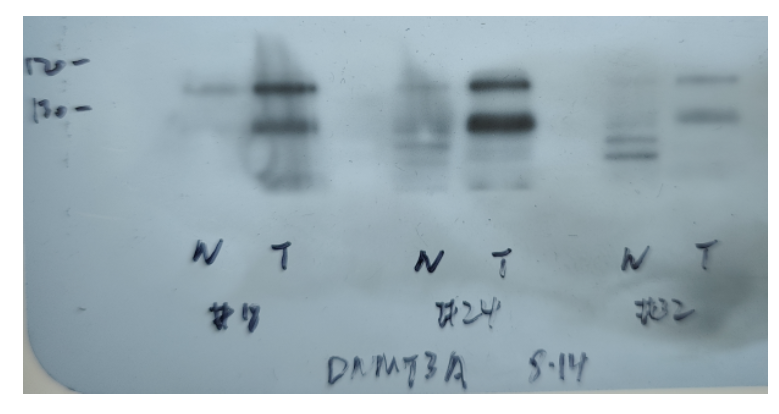

Supplement: Supplementary file 1 [file biomolecules-14-01088-s001.zip › DNMT3A of 1-2.tif]

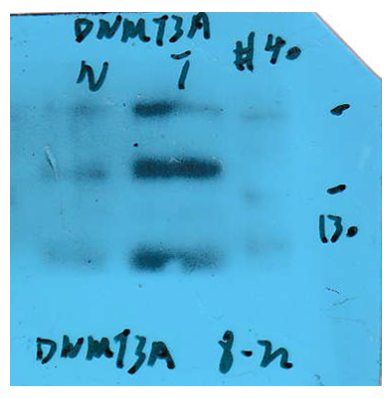

Supplement: Supplementary file 1 [file biomolecules-14-01088-s001.zip › DNMT3A of 10.tif]

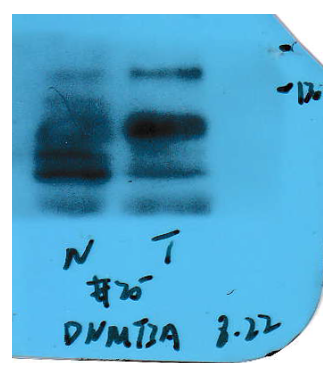

Supplement: Supplementary file 1 [file biomolecules-14-01088-s001.zip › DNMT3A of 11.tif]

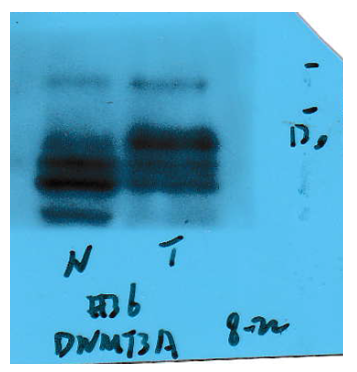

Supplement: Supplementary file 1 [file biomolecules-14-01088-s001.zip › DNMT3A of 12.tif]

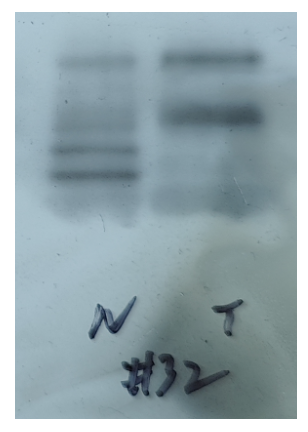

Supplement: Supplementary file 1 [file biomolecules-14-01088-s001.zip › DNMT3A of 3.tif]

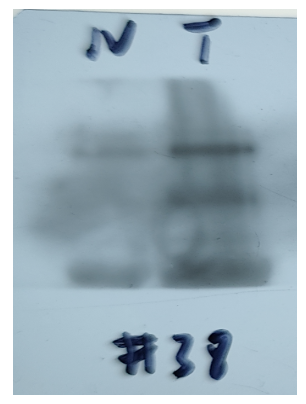

Supplement: Supplementary file 1 [file biomolecules-14-01088-s001.zip › DNMT3A of 4.tif]

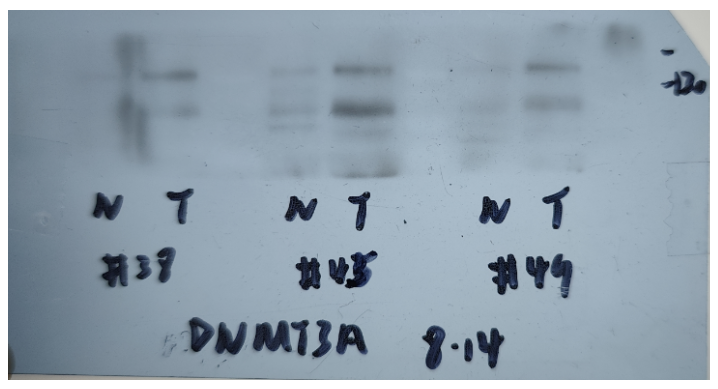

Supplement: Supplementary file 1 [file biomolecules-14-01088-s001.zip › DNMT3A of 5-6.tif]

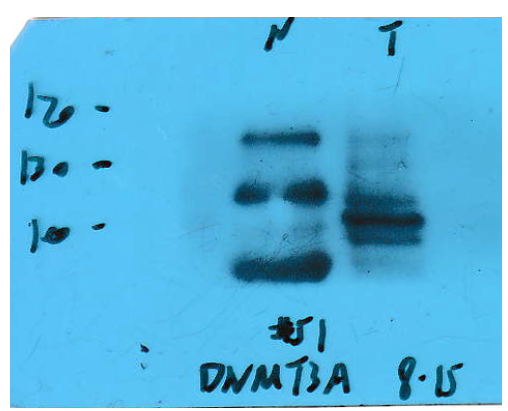

Supplement: Supplementary file 1 [file biomolecules-14-01088-s001.zip › DNMT3A of 7.tif]

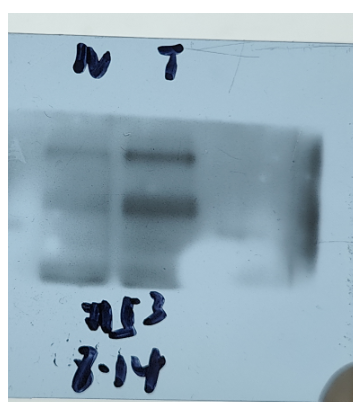

Supplement: Supplementary file 1 [file biomolecules-14-01088-s001.zip › DNMT3A of 8.tif]

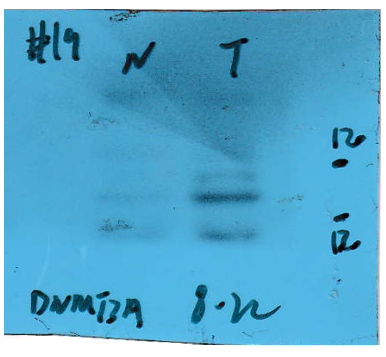

Supplement: Supplementary file 1 [file biomolecules-14-01088-s001.zip › DNMT3A of 9.tif]

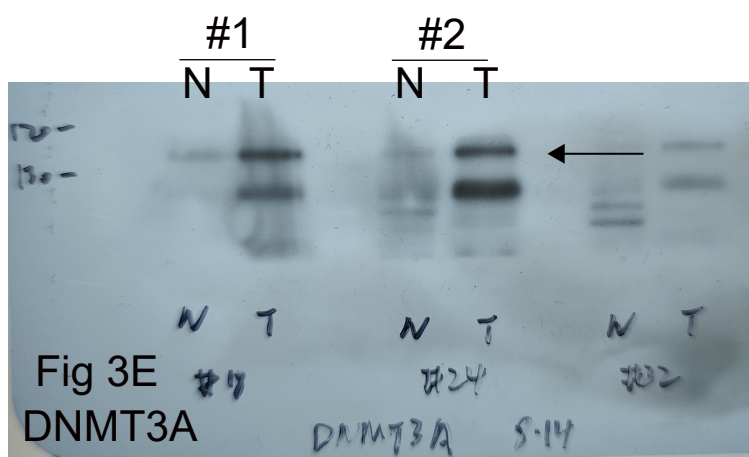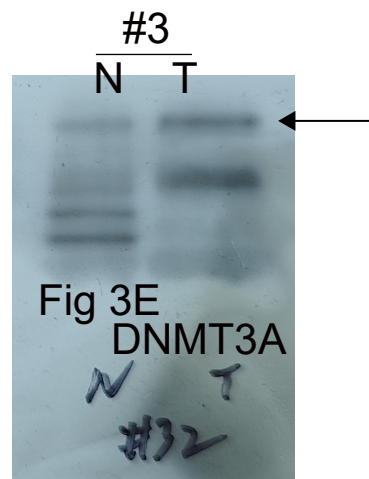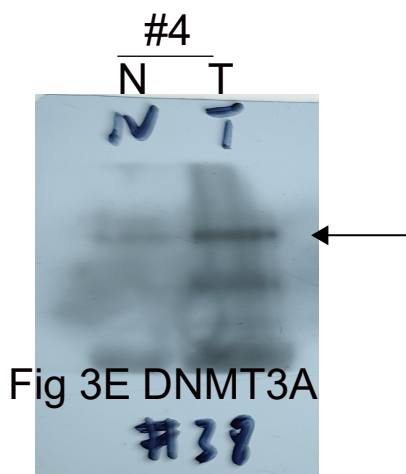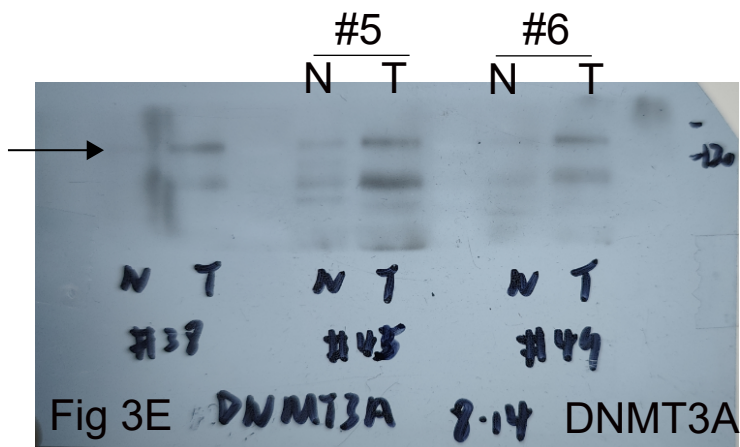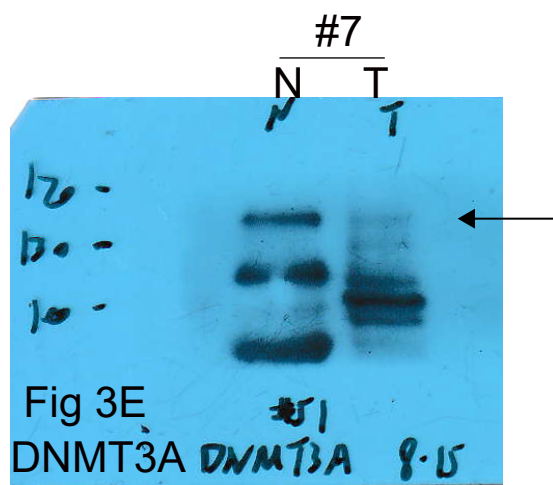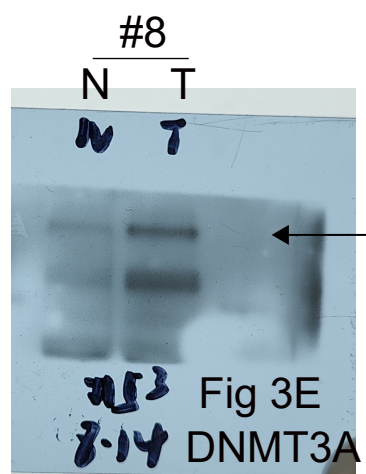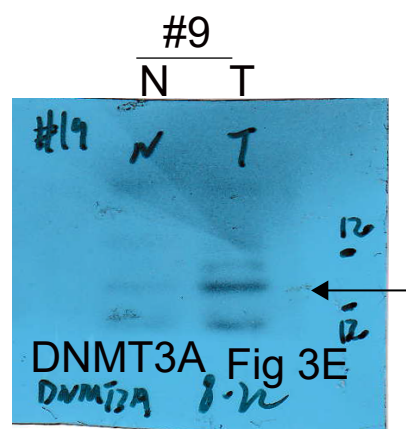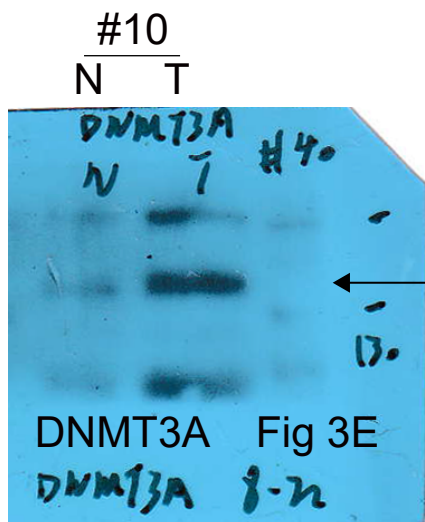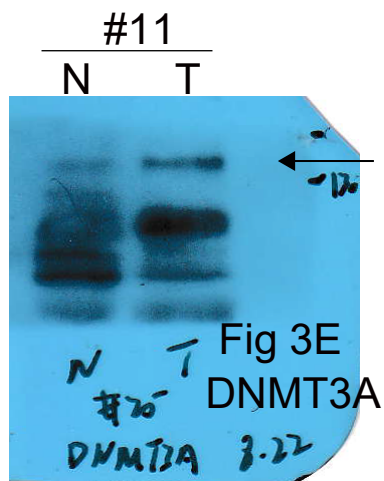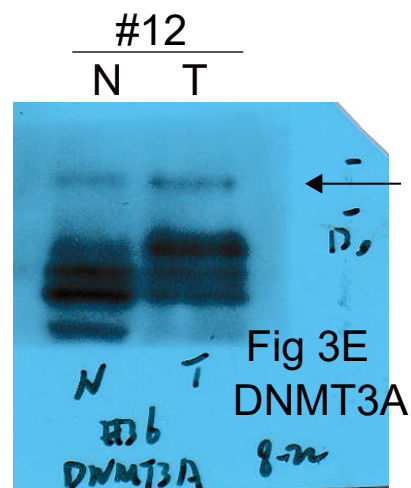

Supplement: Supplementary file 1 [file biomolecules-14-01088-s001.zip › DNMT3A of Fig 3E.pdf]

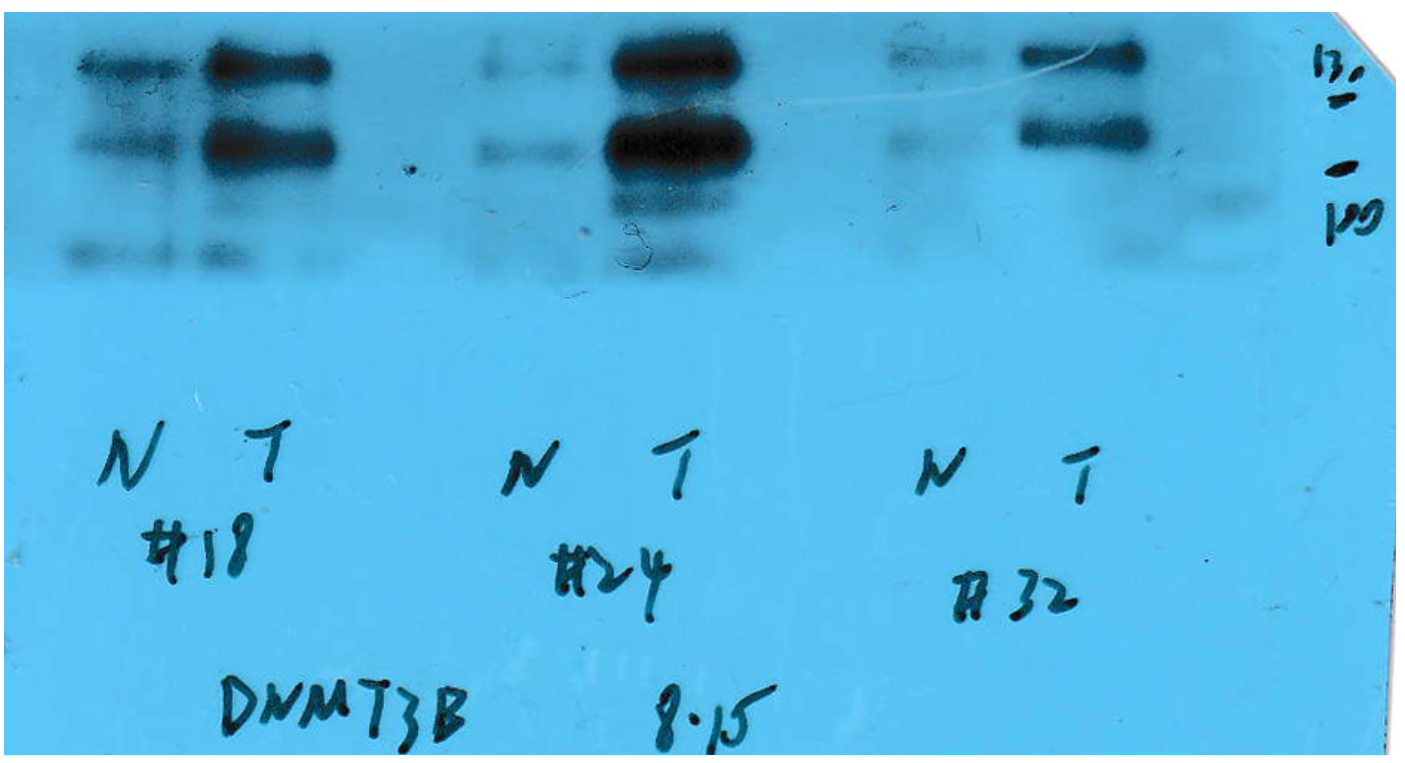

Supplement: Supplementary file 1 [file biomolecules-14-01088-s001.zip › DNMT3B of 1-3.tif]

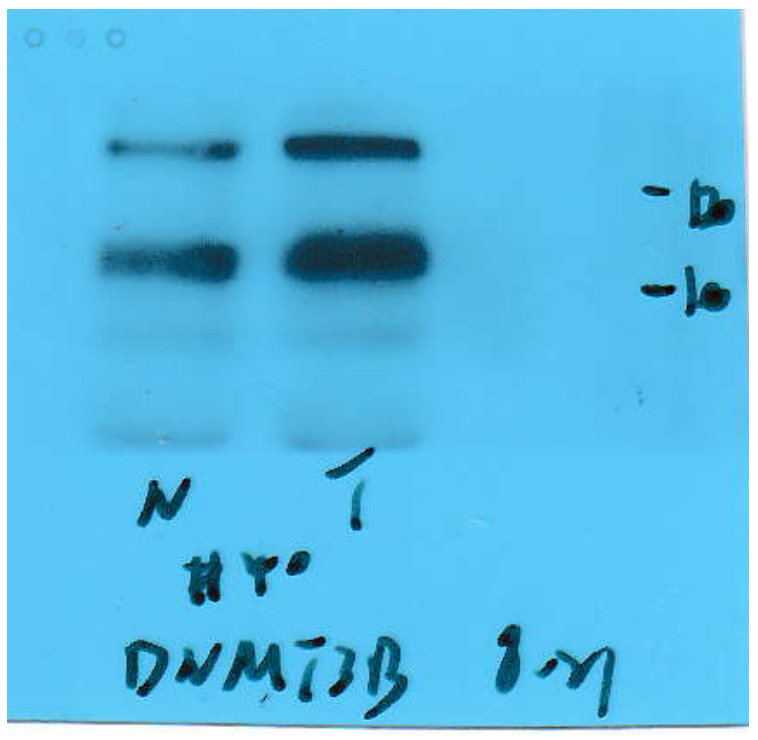

Supplement: Supplementary file 1 [file biomolecules-14-01088-s001.zip › DNMT3B of 10.tif]

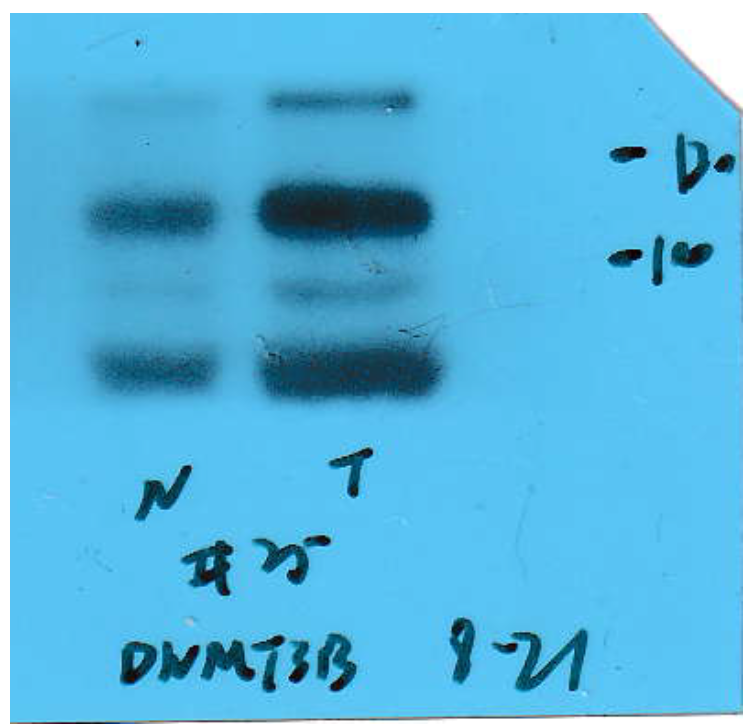

Supplement: Supplementary file 1 [file biomolecules-14-01088-s001.zip › DNMT3B of 11.tif]

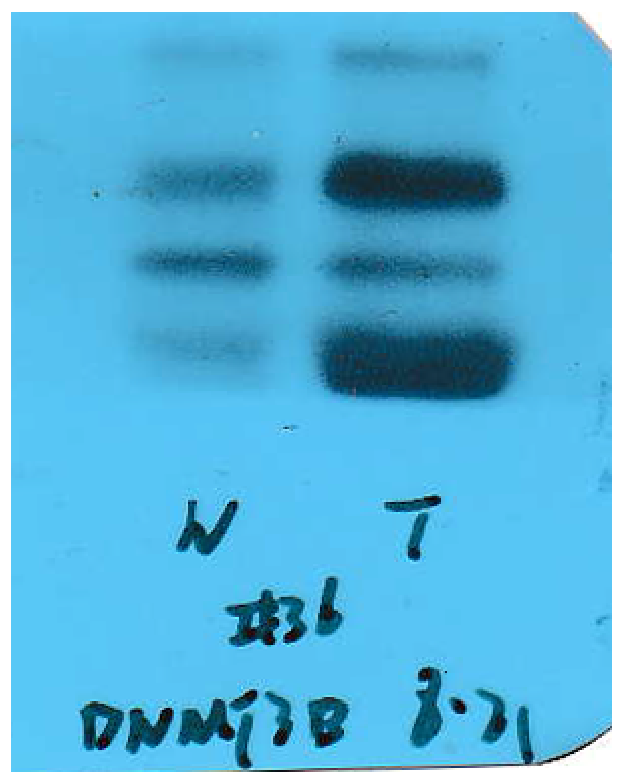

Supplement: Supplementary file 1 [file biomolecules-14-01088-s001.zip › DNMT3B of 12.tif]

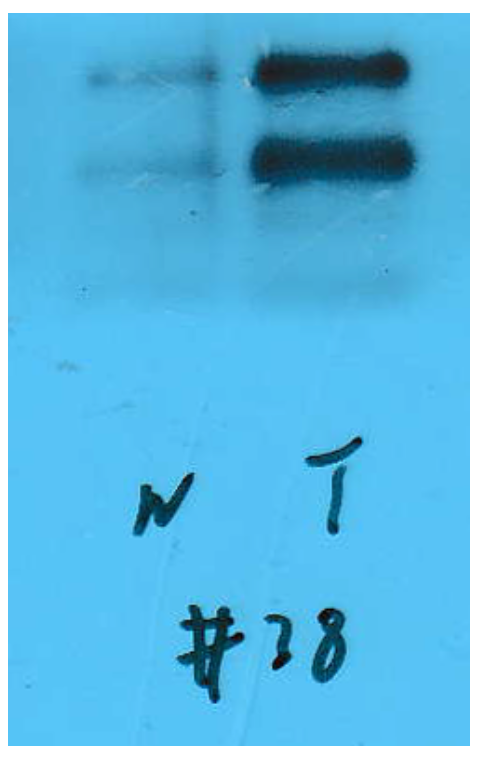

Supplement: Supplementary file 1 [file biomolecules-14-01088-s001.zip › DNMT3B of 4.tif]

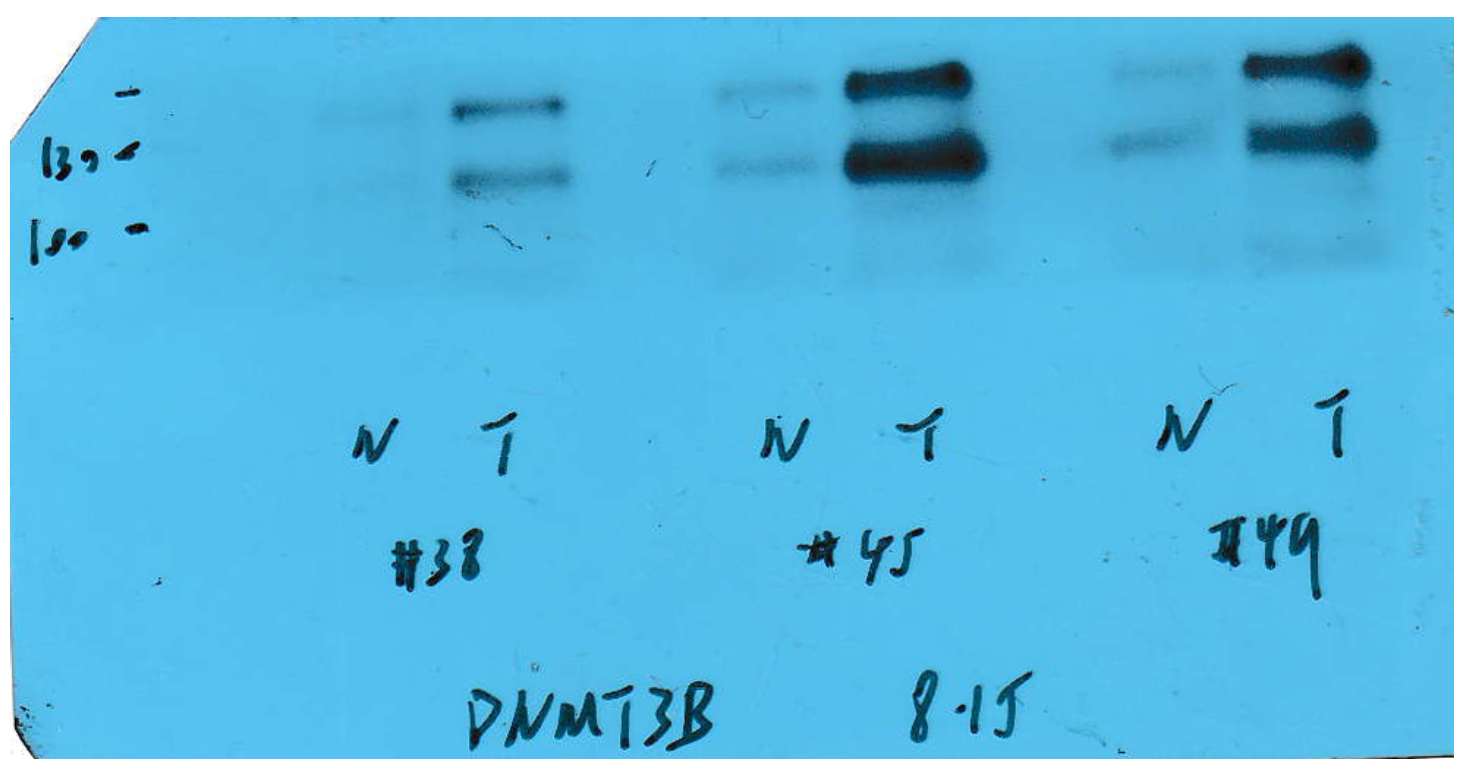

Supplement: Supplementary file 1 [file biomolecules-14-01088-s001.zip › DNMT3B of 5-6.tif]

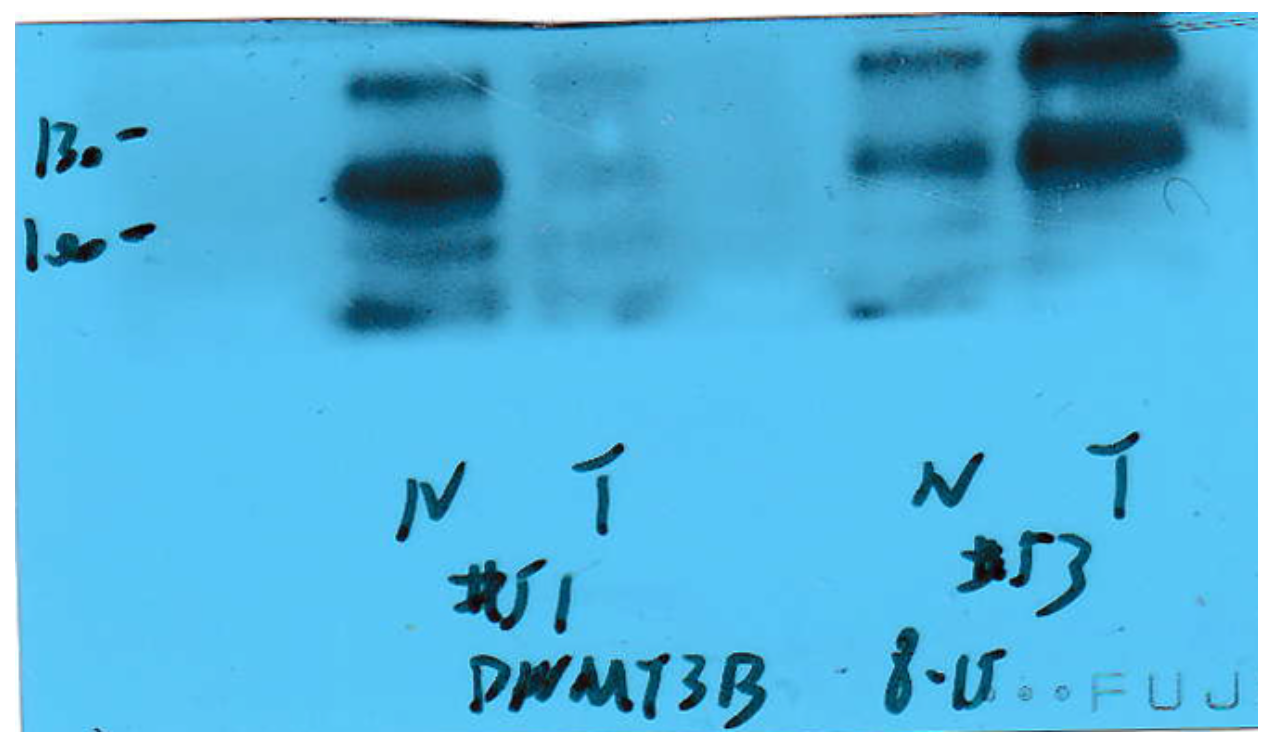

Supplement: Supplementary file 1 [file biomolecules-14-01088-s001.zip › DNMT3B of 7-8.tif]

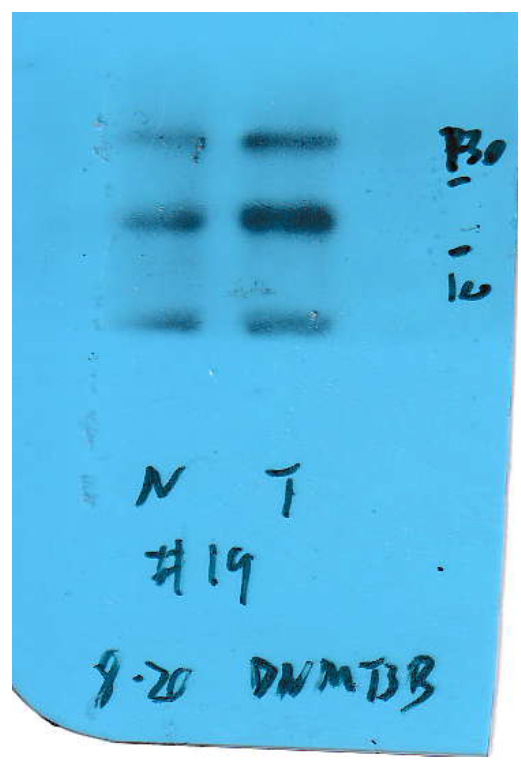

Supplement: Supplementary file 1 [file biomolecules-14-01088-s001.zip › DNMT3B of 9.tif]

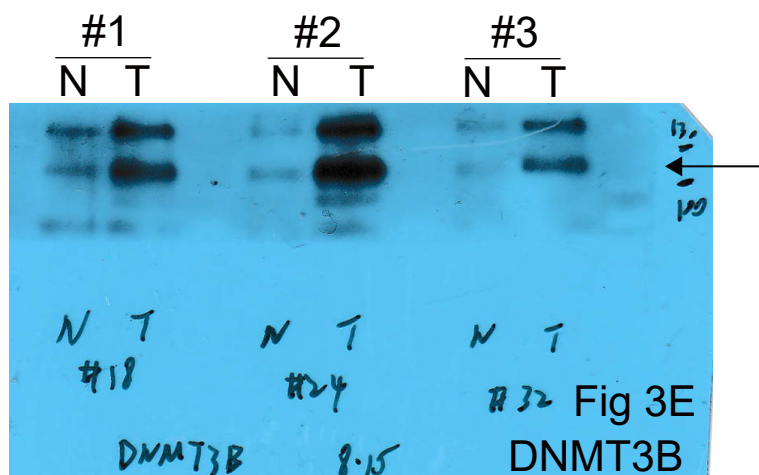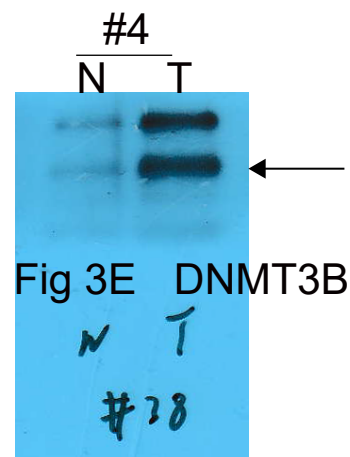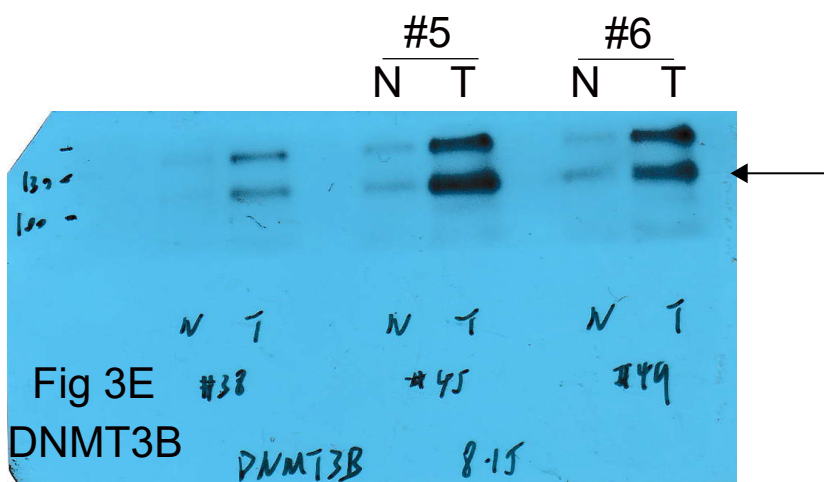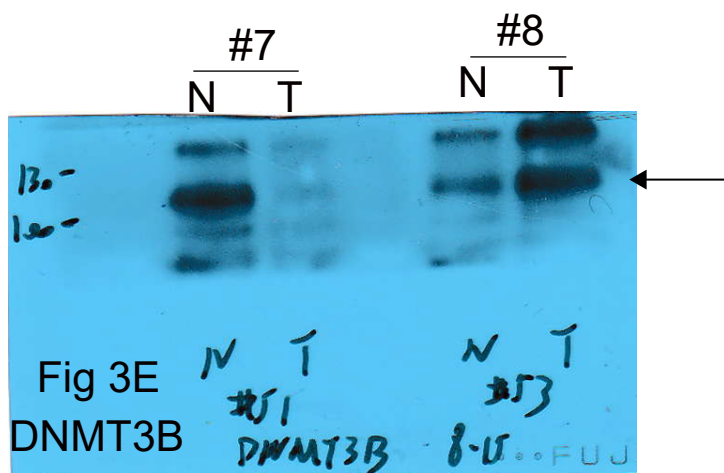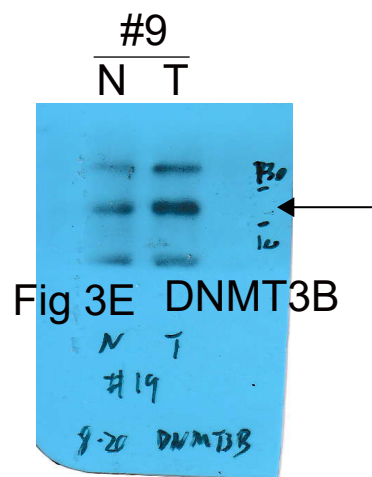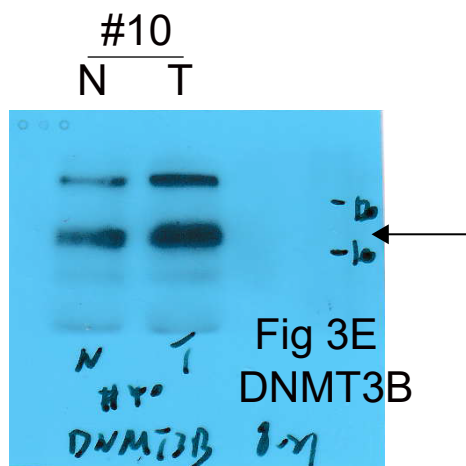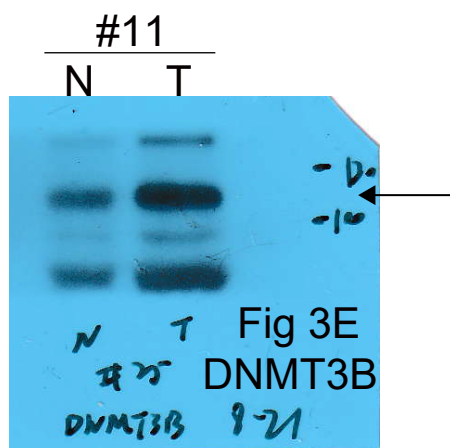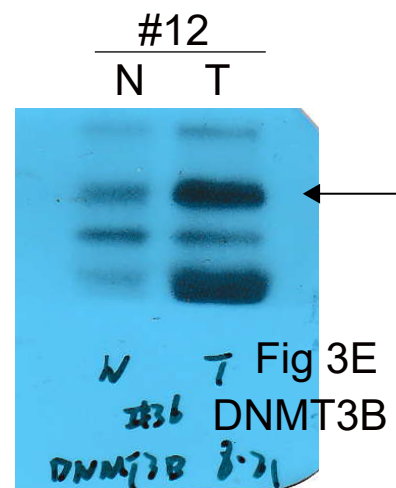

Supplement: Supplementary file 1 [file biomolecules-14-01088-s001.zip › DNMT3B of Fig 3E.pdf]

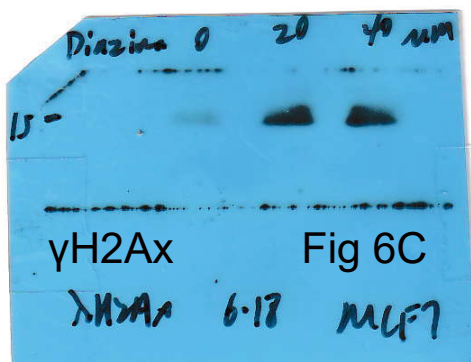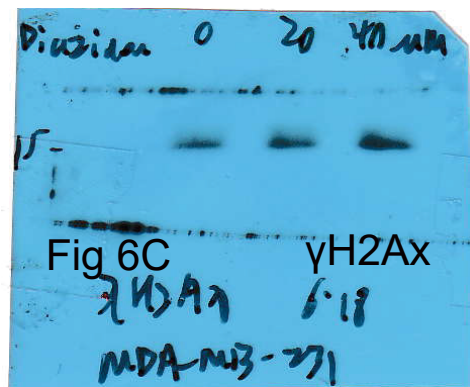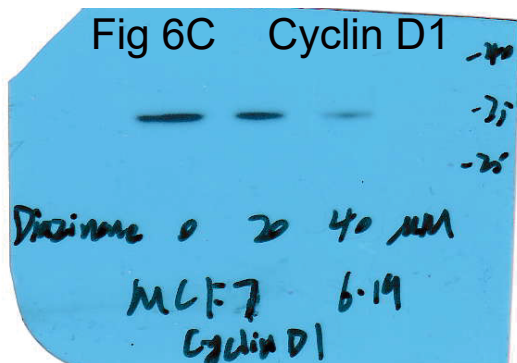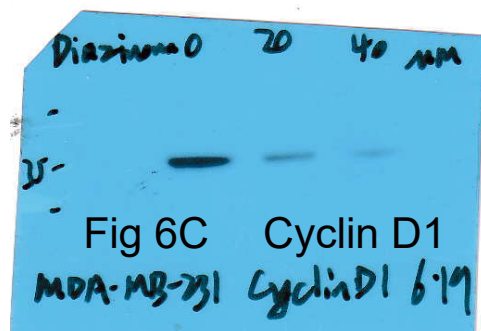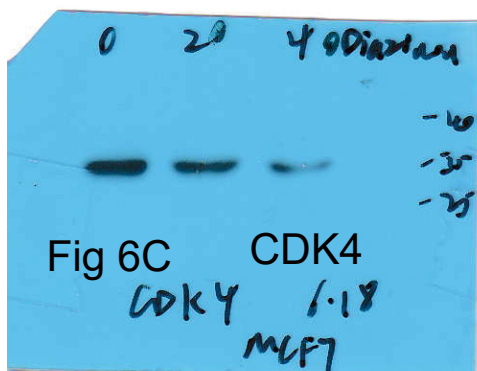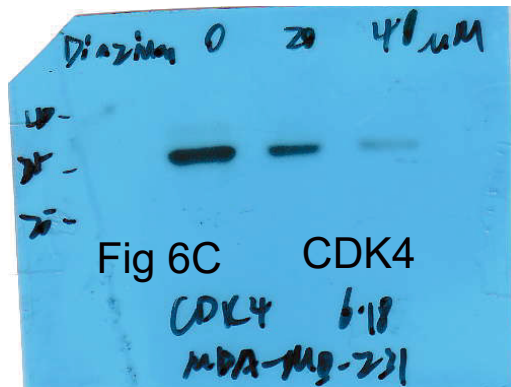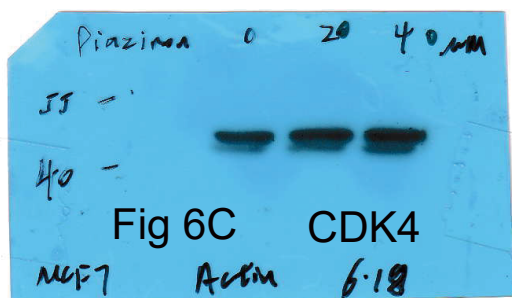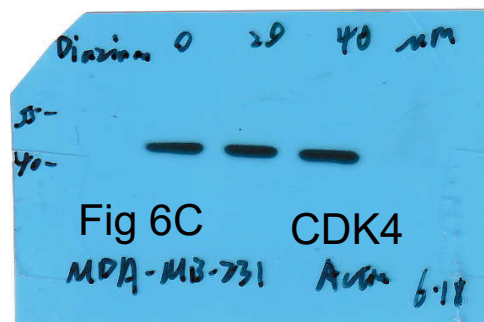

Supplement: Supplementary file 1 [file biomolecules-14-01088-s001.zip › Fig 6C.pdf]

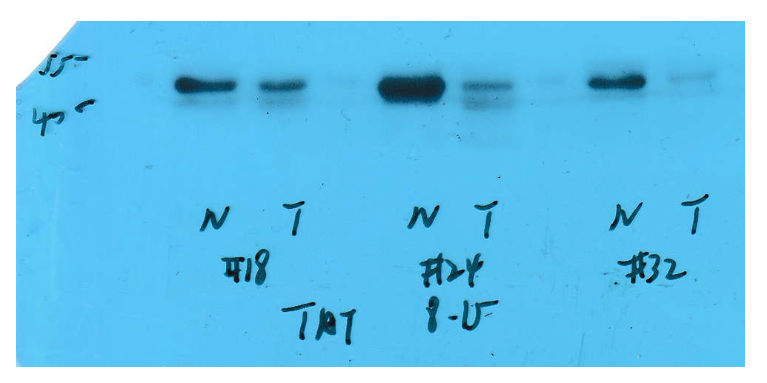

Supplement: Supplementary file 1 [file biomolecules-14-01088-s001.zip › TAT of 1-3.tif]

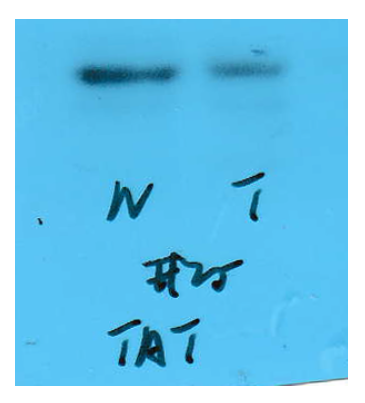

Supplement: Supplementary file 1 [file biomolecules-14-01088-s001.zip › TAT of 11.tif]

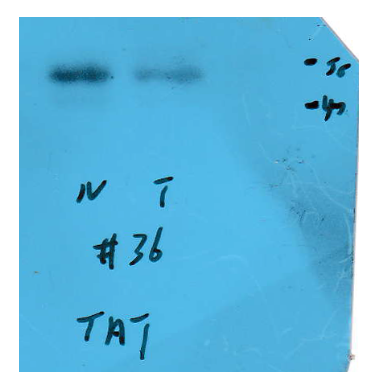

Supplement: Supplementary file 1 [file biomolecules-14-01088-s001.zip › TAT of 12.tif]

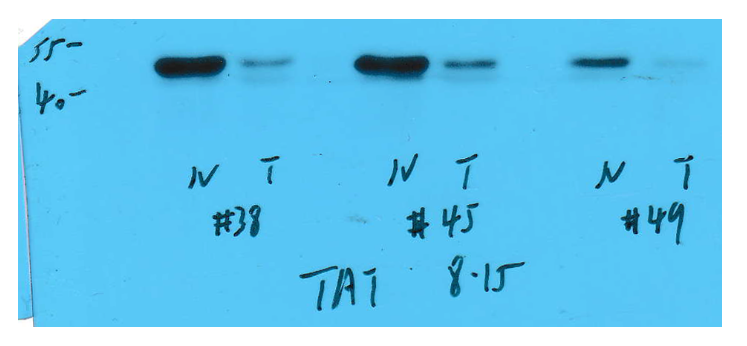

Supplement: Supplementary file 1 [file biomolecules-14-01088-s001.zip › TAT of 4-6.tif]

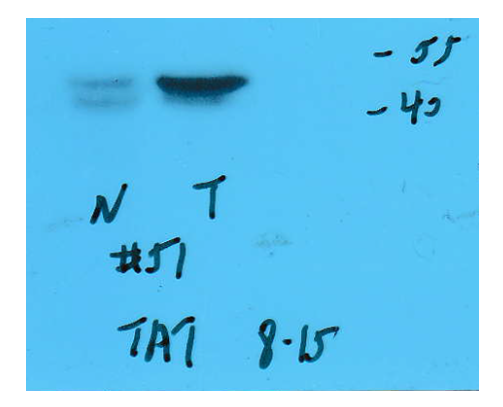

Supplement: Supplementary file 1 [file biomolecules-14-01088-s001.zip › TAT of 7.tif]

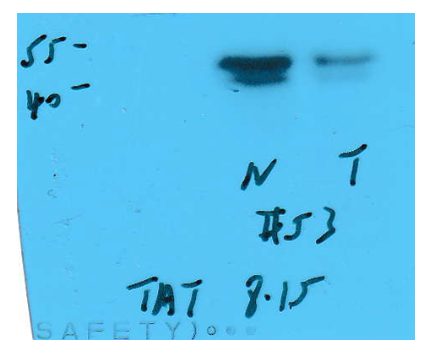

Supplement: Supplementary file 1 [file biomolecules-14-01088-s001.zip › TAT of 8.tif]

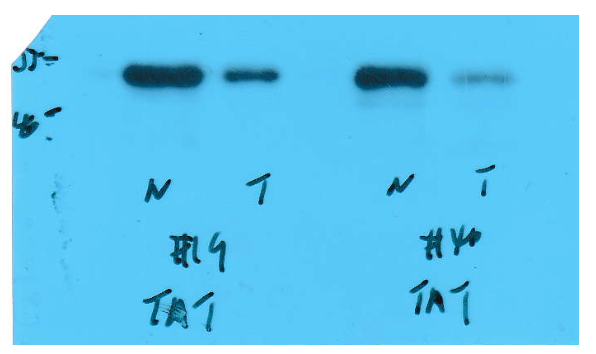

Supplement: Supplementary file 1 [file biomolecules-14-01088-s001.zip › TAT of 9-10.tif]

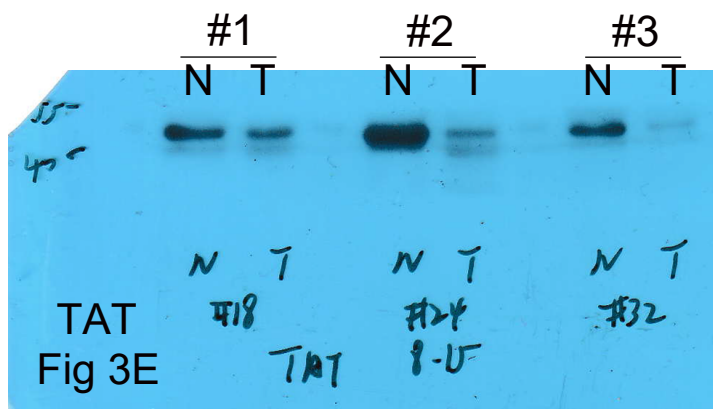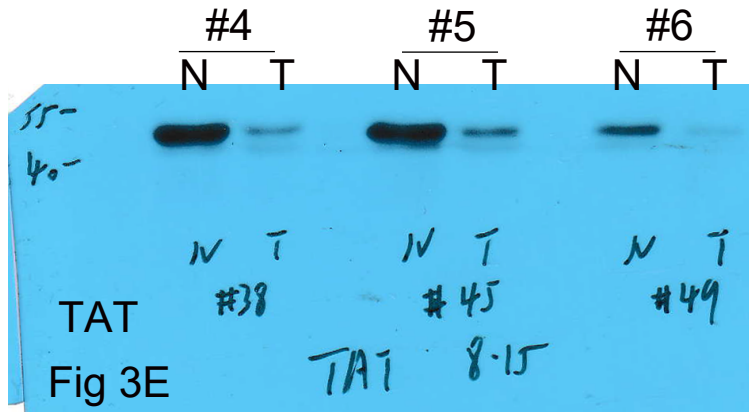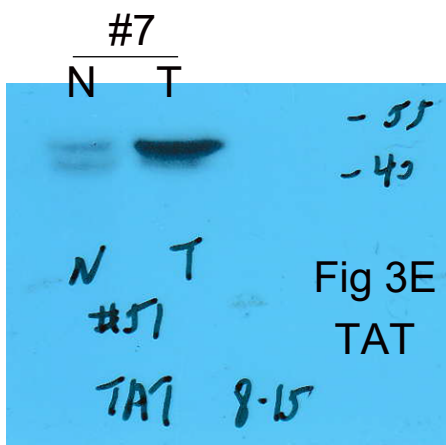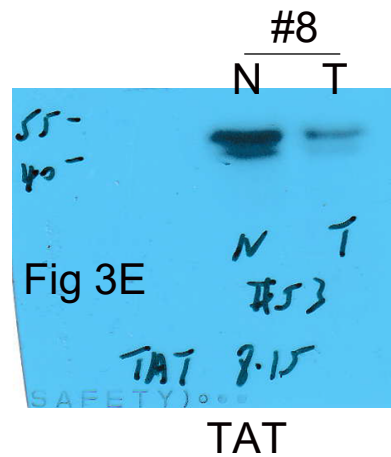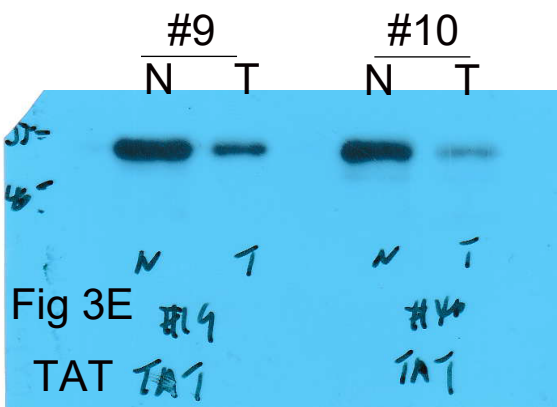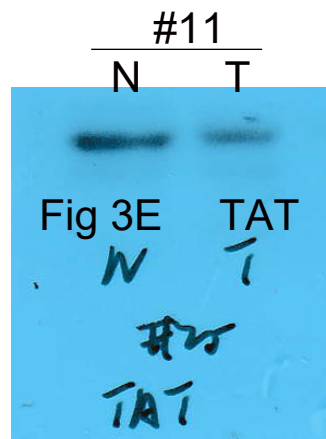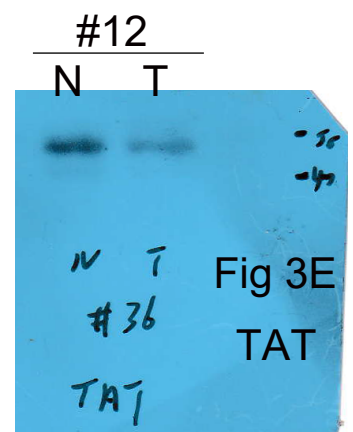

Supplement: Supplementary file 1 [file biomolecules-14-01088-s001.zip › TAT of Fig 3E.pdf]

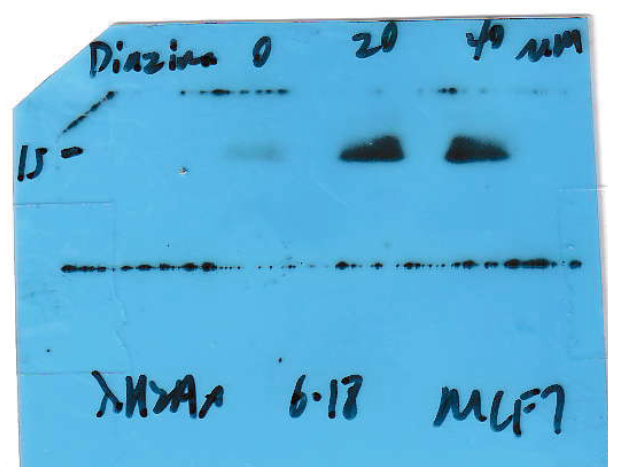

Supplement: Supplementary file 1 [file biomolecules-14-01088-s001.zip › a├H2Ax-MCF7.tif]

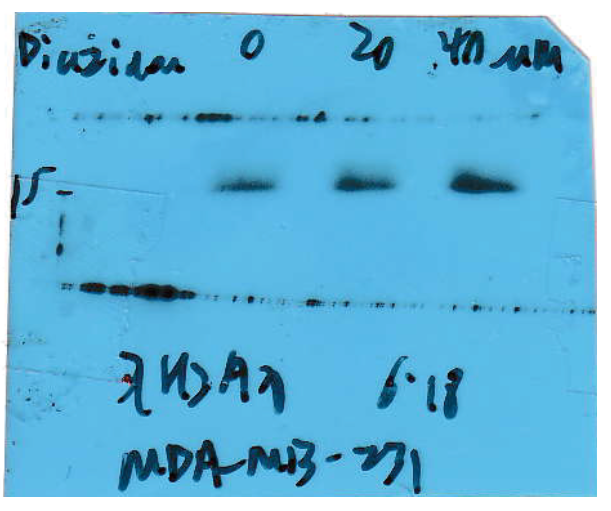

Supplement: Supplementary file 1 [file biomolecules-14-01088-s001.zip › a├H2Ax-MDA-MB-231.tif]
